# Reduced intestinal lipid absorption improves glucose metabolism in aged G2-Terc knockout mice

**DOI:** 10.1186/s12915-023-01629-8

**Published:** 2023-07-04

**Authors:** Xue Liu, Ahmed Elagamy Mohamed Mahmoud Khalil, Uthayakumar Muthukumarasamy, Yasuhiro Onogi, Xiaocheng Yan, Inderjeet Singh, Elena Lopez-Gonzales, Andreas Israel, Alberto Cebrian Serrano, Till Strowig, Siegfried Ussar

**Affiliations:** 1 RG Adipocytes & Metabolism, Institute for Diabetes & Obesity, Helmholtz Diabetes Center, Helmholtz Center Munich, Helmholtz Zentrum München, German Research Center for Environmental Health GmbH, Ingolstaedter Landstrasse 1, 85764 Neuherberg, Germany; 2 German Center for Diabetes Research (DZD), 85764 Neuherberg, Germany; 3 Microbial Immune Regulation Research Group, Helmholtz Centre for Infection Research, Brunswick, Germany; 4 Institute for Diabetes & Obesity, Helmholtz Diabetes Center, Helmholtz Zentrum München, German Research Center for Environmental Health GmbH, 85764 Neuherberg, Germany; 5 Hannover Medical School, Hannover, Germany; 6 Department of Medicine, Technische Universität München, Munich, Germany

**Keywords:** Adipose tissue, Aging, Glucose tolerance, Insulin sensitivity, Lipid absorption, Microbiota, Obesity, Telomerase

## Abstract

**Background:**

Biological aging is an important factor leading to the development of pathologies associated with metabolic dysregulation, including type 2 diabetes, cancer, cardiovascular and neurodegenerative diseases. Telomere length, a central feature of aging, has additionally been identified as inversely associated with glucose tolerance and the development of type 2 diabetes. However, the effects of shortened telomeres on body weight and metabolism remain incompletely understood. Here, we studied the metabolic consequences of moderate telomere shortening using second generation loss of telomerase activity in mice.

**Results:**

Aged male and female G2 Terc-/- mice and controls were characterized with respect to body weight and composition, glucose homeostasis, insulin sensitivity and metabolic activity. This was complemented with molecular and histological analysis of adipose tissue, liver and the intestine as well as microbiota analysis. We show that moderate telomere shortening leads to improved insulin sensitivity and glucose tolerance in aged male and female G2 Terc-/- mice. This is accompanied by reduced fat and lean mass in both sexes. Mechanistically, the metabolic improvement results from reduced dietary lipid uptake in the intestine, characterized by reduced gene expression of fatty acid transporters in enterocytes of the small intestine. Furthermore, G2-Terc-/- mice showed significant alterations in the composition of gut microbiota, potentially contributing to the improved glucose metabolism.

**Conclusions:**

Our study shows that moderate telomere shortening reduces intestinal lipid absorption, resulting in reduced adiposity and improved glucose metabolism in aged mice. These findings will guide future murine and human aging studies and provide important insights into the age associated development of type 2 diabetes and metabolic syndrome.

**Supplementary Information:**

The online version contains supplementary material available at 10.1186/s12915-023-01629-8.

## Background

Aging has pleiotropic effects on organismal function differentially affecting individual organs. Changes in aging associated organ function contributes to the development of various diseases, such as cancer, osteoporosis and neurodegenerative diseases [1]. Metabolically, aging is associated with glucose intolerance [2, 3], the development of type 2 diabetes and metabolic syndrome [4]. Importantly, diet, level of exercise, age-associated changes in gut microbiota and shortening of telomeres are well-established common factors driving age-related diseases including type 2 diabetes and metabolic syndrome [5–7]. Thus, individual lifestyle choices let us modify environmental drivers of age-associated diseases. This in turn changes the impact of individual genetic predispositions on disease development and progression. However, the complex interplay between the environment, gut microbiota and host genetics, and the resulting consequences for aging and physiology remain under ongoing investigation [8, 9]. One example for the strong impact of life-style choices on genetics is the regulation of telomere length. Telomere length is strongly associated with diet and body weight, smoking, physical activity etc. Telomeres shorten with each cell division and short telomeres are an indicator of premature biological aging [10, 11]. When telomeres become critically short, cells go into senescence or apoptosis due to chromosomal instability [7]. To counteract this process, certain cell types such as stem cells, germ cells, certain somatic cells like lymphocytes and intestinal epithelial cells [12, 13] use telomerase [14]. This ribonucleoprotein enzyme complex elongates telomeres and maintains proliferative capacity. Telomerase consists of the telomerase reverse transcriptase (Tert) and the telomerase RNA component (Terc), which acts as a template for the synthesis of telomeres by Tert [15, 16].

Telomere length and the development of type 2 diabetes and metabolic syndrome are regulated by similar environmental factors. Thus, it is not surprising that shorter telomeres are associated with glucose intolerance and type 2 diabetes [17, 18] and an elevated risk for developing metabolic syndrome [19]. In line with a direct effect of telomere length with the development of type 2 diabetes and metabolic syndrome, Terc knockout mice inbred for four generations showed impaired insulin secretion from pancreatic beta cells and impaired systemic glucose homeostasis [20]. However, the interpretation of these data is complicated by the severe pathology and early lethality of fourth generation Terc knockout mice (G4-Terc^−/−^). Conversely, aged G3-Terc^−/−^ mice showing reduced life expectancy but a less severe phenotype, had reduced body weight and increased glucose metabolism that was normalized by feeding a high sugar diet [21]. To this end, it remains unclear how shortened telomeres interfere with glucose and insulin homeostasis as well as body weight regulation.

Here we performed a detailed metabolic characterization of aged female and male G2-Terc^−/−^ mice to obtain mechanistic insights into the impact of accelerated telomere shortening on glucose metabolism in geriatric subjects. Our data in G2-Terc^−/−^ female and male mice demonstrate a beneficial effect of shortened telomeres on insulin sensitivity and glucose homeostasis in both sexes. In addition, G2-Terc^−/−^ mice showed significant alterations in the composition of gut microbiota with a specific increase in *Bifidobacteriaceae* and *Erysipelotrichaceae*. This beneficial effect is likely driven by reduced intestinal lipid absorption characterized by reduced expression of fatty acid transporters in enterocytes. Thus, moderate acceleration of telomere shortening in aged mice appears to reduce intestinal lipid uptake, resulting in lower adiposity, reduced liver triglyceride levels and an overall improved glucose tolerance and insulin sensitivity.

## Results

**Fig. 1:**
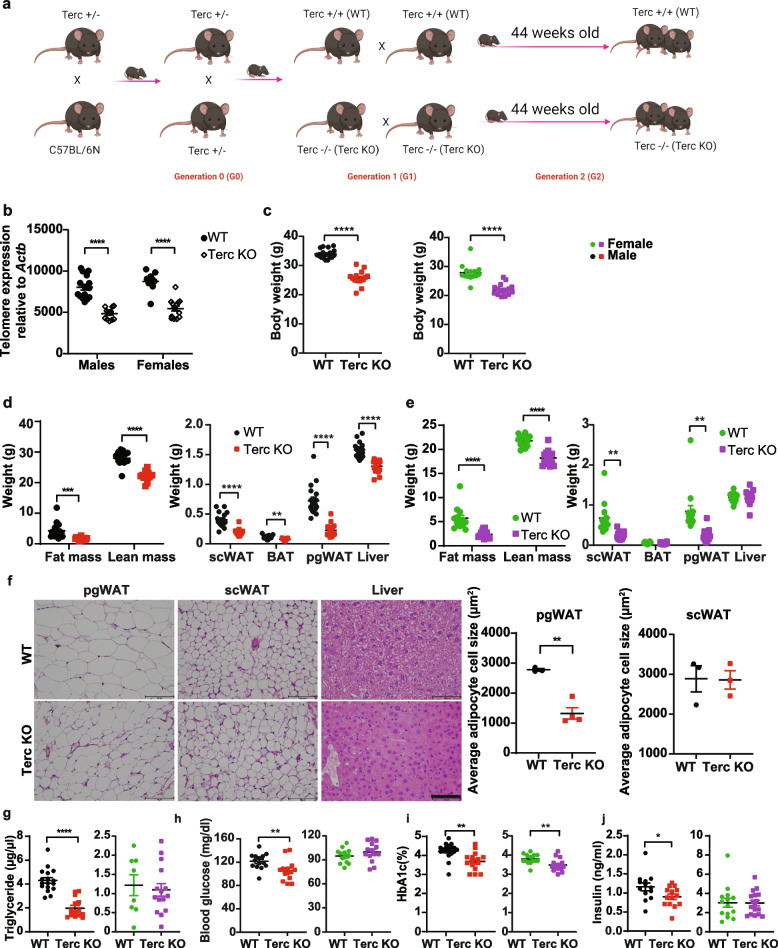
Telomere shortening and metabolic characterization of G2-Terc^−/−^ mice. **a** Breeding scheme for second generation Terc KO mice. **b** Telomere length relative to *Actb* in male and female enterocytes (male *n* = 15 WT, *n* = 12 KO; female *n* = 8 WT, *n* = 14 KO, 14 months old). **c** Body weight, (**d**,**e**) Body composition and tissue weights at 14 months of age. male *n* = 18 WT, *n* = 15 KO (**d**), female *n* = 14 WT, *n* = 16 KO (**e**). **f** H&E stainings of perigonodal white adipose tissue (pgWAT), subcutaneous white adipose tissue (scWAT) and liver of male G2-Terc^−/−^ mice and controls and adipocyte cell size quantification in pgWAT and scWAT. Scale bar shows 100 µm. Adipocyte cell size quantification raw data are included in Additional file 3: Table S1a. **g** Serum triglyceride levels in 14 month old mice (male *n* = 17 WT, *n* = 14 KO; female *n* = 8 WT, *n* = 15 KO). **h** Fasting glycemia in 12 months old mice (male *n* = 12 WT, *n* = 14 KO; female *n* = 14 WT, *n* = 16 KO). **i** Percentage of glycosylated hemoglobin (HbA1c%) in 13 months old mice (male *n* = 18 WT, *n* = 15 KO; female *n* = 14 WT, *n* = 16 KO). **j** Fasting serum insulin levels in 12 month old mice (male *n* = 13 WT, *n* = 14 KO; female *n* = 14 WT, *n* = 16 KO). All data are shown as mean ± SE. Statistical analysis was performed using an unpaired *t*-test or ordinary two-way ANOVA with Sidak’s multiple comparison test. *p < 0.05, **p < 0.01, ***p < 0.001, ****p < 0.0001. Red and black dots represent male mice. Green and purple dots represent female mice

To study the metabolic effects of telomerase deficiency without the increased mortality observed from third generation Terc^−/−^ mice onwards, we studied aged (41 weeks- > 10 months old) second generation Terc^−/−^ inbred mice (G2-Terc^−/−^, Fig. 1a). Wild type (WT) controls were derived from the same breedings of Terc^±^ mice and inbred as a separate cohort in parallel to Terc^−/−^ (Terc KO) mice (Fig. 1a). Analysis of intestinal epithelial telomere length of 14 months old G2-Terc^−/−^ mice confirmed a 40% reduction in male and 38% reduction in female telomere length compared to age matched control mice (unpaired *t*-test, *p* < 0.0001, Fig. 1b). Similar to previous observations in G3-Terc^−/−^ mice [21], body weight of both male and female G2-Terc^−/−^ mice was significantly lower compared to G2-Terc^+/+^ controls (unpaired *t-*test, *p* < 0.0001, male: WT 34 ± 1.6 g; ko 25.7 ± 2.5 g; female: WT 27.9 ± 2.9 g; ko 22 ± 1.9 g, Fig. 1c). Reduced body weight, largely dependent on reduced lean mass, was already observed in nine weeks old G2-Terc^−/−^ male mice (unpaired *t-*test, male *p* < 0.0001, WT 26 ± 1.5 g; ko 24 ± 1.4 g, Additional file 1: Figure S1a;) but not in nine weeks old female mice (unpaired *t-*test, *p* = 0.977, Additional file 1: Figure S1b). In line with the reduced body weight, aged G2-Terc^−/−^ mice had reduced lean and fat mass (unpaired *t*-test between genotype, male: fat mass *p* = 0.0001, WT 4.3 ± 2.4 g, ko 1.7 ± 0.5 g; lean mass *p* < 0.0001, WT 28 ± 2 g, ko 22 ± 1.5 g; female: lean mass WT 21.8 ± 1.2, ko 18.2 ± 1.4 g and fat mass WT 5.7 ± 2.3 g, ko 2.3 ± 0.7 g, *p* < 0.0001; Fig. 1d and e). The weight of subcutaneous white adipose tissue (scWAT), brown adipose tissue (BAT), perigonadal WAT (pgWAT) and liver was significantly lower in male G2-Terc^−/−^ mice with 53%,29%, 69% and 17% reduction compared to controls, respectively (unpaired *t*-test between genotypes in each tissue, male: BAT *p* = 0.0009, scWAT, pgWAT and liver *p* < 0.0001; Fig. 1d). Female G2-Terc^−/−^ mice showed a 63% and 69% reduction in scWAT and pgWAT mass, respectively, while BAT and liver mass were not different from control mice (unpaired *t*-test between genotypes in each tissue female scWAT *p* < 0.0001, pgWAT *p* < 0.0001, BAT and liver *p* > 0.05; Fig. 1e). Histological analysis of pgWAT, scWAT and liver in male mice revealed a 52% reduction in adipocyte size in pgWAT (unpaired t-test, *p* = 0.0227) without any differences in subcutaneous adipocyte size in G2-Terc^−/−^ mice compared to controls (Fig. 1f). Liver histology suggested reduced hepatic triglyceride levels, which were observed in female but not male mice (Additional file 1; Figure S1c). Triglyceride levels were also not different in the tibialis anterior (TA), albeit a trend for an increase in female mice was observed (Additional file 1:Figure S1c). Oxidative phosphorylation (Oxphos) protein levels were not different between control and G2-Terc^−/−^ mice in liver and TA in both male and female mice (Additional file 1; Figure S1d). Gene expression of IL-6 and TNFα was reduced by 41% and 29% in pgWAT of male G2-Terc^−/−^ mice, respectively (unpaired *t-*test, Il6 *p* = 0.0037; TNFα *p* = 0.0504, Additional file 1; Figure S1e and f). Male G2-Terc^−/−^ mice also showed 50% lower serum triglyceride levels (unpaired *t-*test, *p* < 0.0001; Fig. 1g), reduced fasting glucose (unpaired *t-*test, *p* = 0.0040; 11% reduced, Fig. 1h), glycosylated hemoglobin (HbA1c%; unpaired *t-*test, *p* = 0.0012; 13% reduced, Fig. 1i) and fasting insulin levels (unpaired *t-*test, *p* = 0.0415; 22% reduced, Fig. 1j). In contrast to this, female G2-Terc^−/−^ mice showed an 8% reduction in HbA1c% levels (unpaired *t-*test, *p* = 0.0074) relative to controls, but no differences in serum triglycerides and fasting glucose and insulin levels (Fig. 1g-j). In contrast, we did not observe any significant differences in HbA1c% levels of 9 weeks old female and male G2-Terc^−/−^ mice compared to controls (Additional file 1: Figure S1g and h). These data suggest that moderate telomere shortening in aged, but not young mice improves glucose tolerance and insulin sensitivity.

### G2-Terc^−/−^ mice show improved glucose and insulin tolerance

**Fig. 2:**
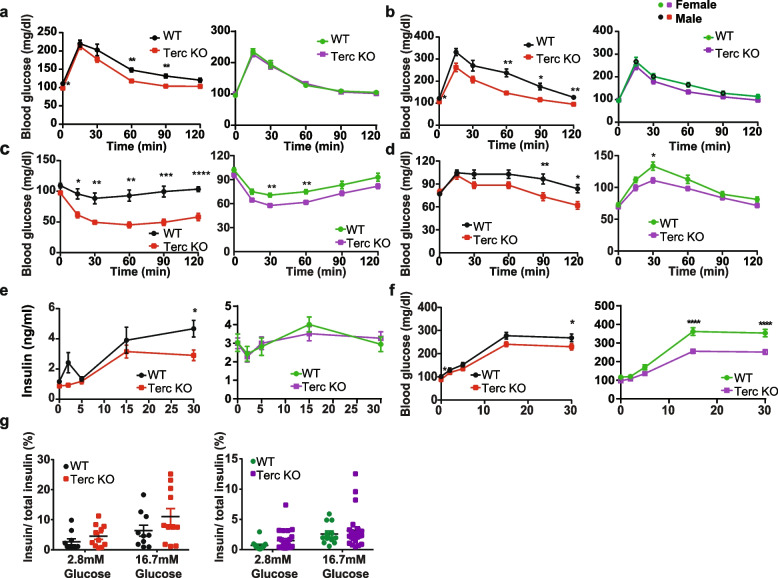
G2-Terc^−/−^ mice show improved insulin sensitivity and glucose tolerance. **a** Oral gavage glucose tolerance test (oGTT) in 12 months old male and female G2-Terc^−/−^ and control mice (male *n* = 12 WT, *n* = 14 KO; female *n* = 14 WT, *n* = 14 KO). **b** Intraperitoneal glucose tolerance test (ipGTT) in 12 months old male and female G2-Terc^−/−^ and control mice (male *n* = 12 WT, *n* = 14 KO; female *n* = 14 WT, *n* = 14 KO). **c** Insulin tolerance test (ITT) in 12 months male and female G2-Terc^−/−^ and control mice (male *n* = 12 WT, *n* = 12 KO; female *n* = 12 WT, *n* = 14 KO). **d** Pyruvate tolerance test (PTT) in 13 months male and female G2-Terc^−/−^ and control mice (male *n* = 12 WT, *n* = 11 KO; *n* = 14 WT, *n* = 13 KO). **e**,**f** Serum insulin (**e**) and glucose levels (**f**) during an oral glucose stimulated insulin secretion test (oGSIS) performed in 12 months old male and female G2-Terc^−/−^ and control mice (male *n* = 12 WT, *n* = 13 KO, female *n* = 14 WT, *n* = 14 KO). **g** Ex vivo glucose-stimulated insulin secretion test in isolated pancreatic islets from 14 month old male and female G2-Terc^−/−^ and control mice. Percentage of secreted insulin compared to total insulin content. Ten islets were used per well. Male:10 technical replicates from five WT mice and 11 technical replicates from five G2-Terc^−/−^ mice. Female: 12 technical replicates from four WT mice and 24 technical replicates from five G2-Terc^−/−^ mice. All data are shown as mean ± SE. Statistical analysis was performed using a two-way ANOVA followed by a Sidak’s multiple comparison test. *p < 0.05, **p < 0.01, ***p < 0.001, ****p < 0.0001. Red and black dots represent male mice. Green and purple dots represent female mice

To test this hypothesis, we assessed glucose and insulin tolerance as well as oral glucose-stimulated insulin secretion (oGSIS) in 12 months old G2-Terc^−/−^ mice. Moderate telomere shortening led to an improved oral glucose tolerance in male (two-way ANOVA, genotype factor *p* = 0.0066) but not female G2-Terc^−/−^ mice upon oral gavage of 2 g/kg glucose (Fig. 2a). Similar differences were observed by intraperitoneal glucose injection, suggesting no contribution of differences in intestinal glucose absorption to this phenotype (Fig. 2b). Insulin sensitivity was increased in both sexes (two-way ANOVA, genotype factor male *p* < 0.0001, female *p* = 0.0022; Fig. 2c). A pyruvate tolerance test (PTT) in overnight fasted male and female G2-Terc^−/−^ mice revealed a moderately increased disposal of de novo generated glucose in both genders of G2-Terc^−/−^ mice (male: 2-way ANOVA, genotype factor *p* = 0.0424; female: two-way ANOVA; genotype factor *p* = 0.0192 Fig. 2d), largely confirming the increased glucose tolerance and insulin sensitivity. Previous data suggested a direct effect of Terc deficiency on pancreatic beta cell mass and function [20]. To this end, we tested glucose stimulated insulin secretion in response to oral administration of 4 g/kg glucose. Male (two-way ANOVA; genotype factor *p* = 0.0148) but not female G2-Terc^−/−^ mice showed reduced insulin secretion (Fig. 2e). Interestingly, at the higher glucose dose used for the oGSIS compared to the oral glucose tolerance test (oGTT), male (two-way ANOVA, genotype factor *p* = 0.023) and female (two-way ANOVA, genotype factor p *p* < 0.0001) G2-Terc^−/−^ mice showed an increased glucose disposal (Fig. 2f). This indicates that the reduced insulin secretion in G2-Terc^−/−^ mice is the consequence of systemically improved insulin sensitivity and not an impairment in beta cell function. Assessment of insulin secretion from pancreatic beta cells ex vivo did not reveal any statistically significant differences between either male or female G2-Terc^−/−^ mice and controls (Fig. 2g). Taken together, these data suggest that both male and female G2-Terc^−/−^ mice show improved insulin sensitivity and glucose tolerance compared to age matched control mice. However, female mice were more insulin sensitive in general. Thus, significant differences between the genotypes were only detectable upon high glucose doses.

### Telomere shortening does not affect energy expenditure or food intake

**Fig. 3:**
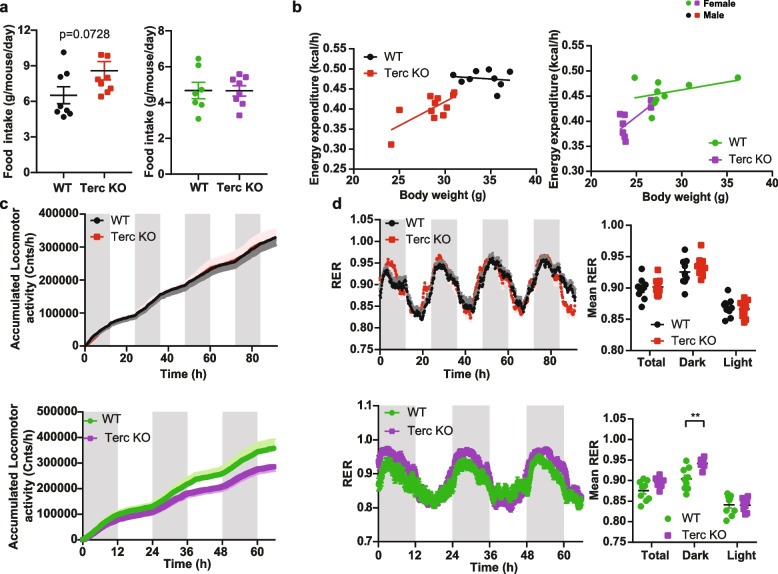
Telomere shortening does not affect energy expenditure or food intake in G2-Terc^−/−^ mice. **a** Daily food intake of 12 month old male and female G2-Terc^−/−^ and control mice (male *n* = 8 WT, *n* = 9 KO; female *n* = 7 WT, *n* = 8 KO). **b**-**d** Metabolic cage measurements of 12-month-old male and female G2-Terc^−/−^ and control mice (male *n* = 9 WT, *n* = 11 KO; female *n* = 9 WT, *n* = 8 KO) Grey areas indicate dark cycle and white areas the light cycle. **b** Correlation of energy expenditure to body weight. **c** Cumulative locomotor activity. **d** Respiratory exchange ratio (RER) and mean total, dark and light cycle RER. All data are shown as mean ± SE. For **a** statistical analysis was performed using an unpaired *t-*test. Statistical analysis for the energy expenditure against body weight was analyzed by ANCOVA. A two-way ANOVA with Sidak’s multiple comparison test was used for **c** and multiple t-tests for **d**. Red and black dots represent male mice. Green and purple dots represent female mice

The increased insulin sensitivity could be a consequence of the reduced fat mass of G2-Terc^−/−^ mice. Thus, we characterized energy balance in metabolic cages. We did not observe differences in food intake or energy expenditure in either male or female G2-Terc^−/−^ mice compared to controls (Fig. 3a-b). We also did not detect significant difference in cumulative locomotor activity in male G2-Terc^−/−^ mice compared to controls, whereas female G2-Terc^−/−^ mice had significantly lower cumulative locomotor activity (Fig. 3c). The respiratory exchange ratio (RER) was not affected by the genotype in male G2-Terc^−/−^ mice (Fig. 3d), whereas female G2-Terc^−/−^ mice showed an increased RER during the dark phase (Fig. 3d, 2-way ANOVA, *p* = 0.0085). Notably, female mice showed genotype specific differences in RER and locomotor activity. However, as this is not observed in male mice, we hypothesized that additional mechanisms shared between genders should exist that significantly contribute to the observed reduction in body weight and insulin sensitivity. Male G2-Terc^−/−^ mice tended to eat more than control mice and energy expenditure tended to decrease in both sexes, which could suggest decreased intestinal nutrient uptake.

### Shorter telomere length results in reduced expression of genes related to fatty acid uptake and a reconfiguration of the gut microbiome

**Fig. 4:**
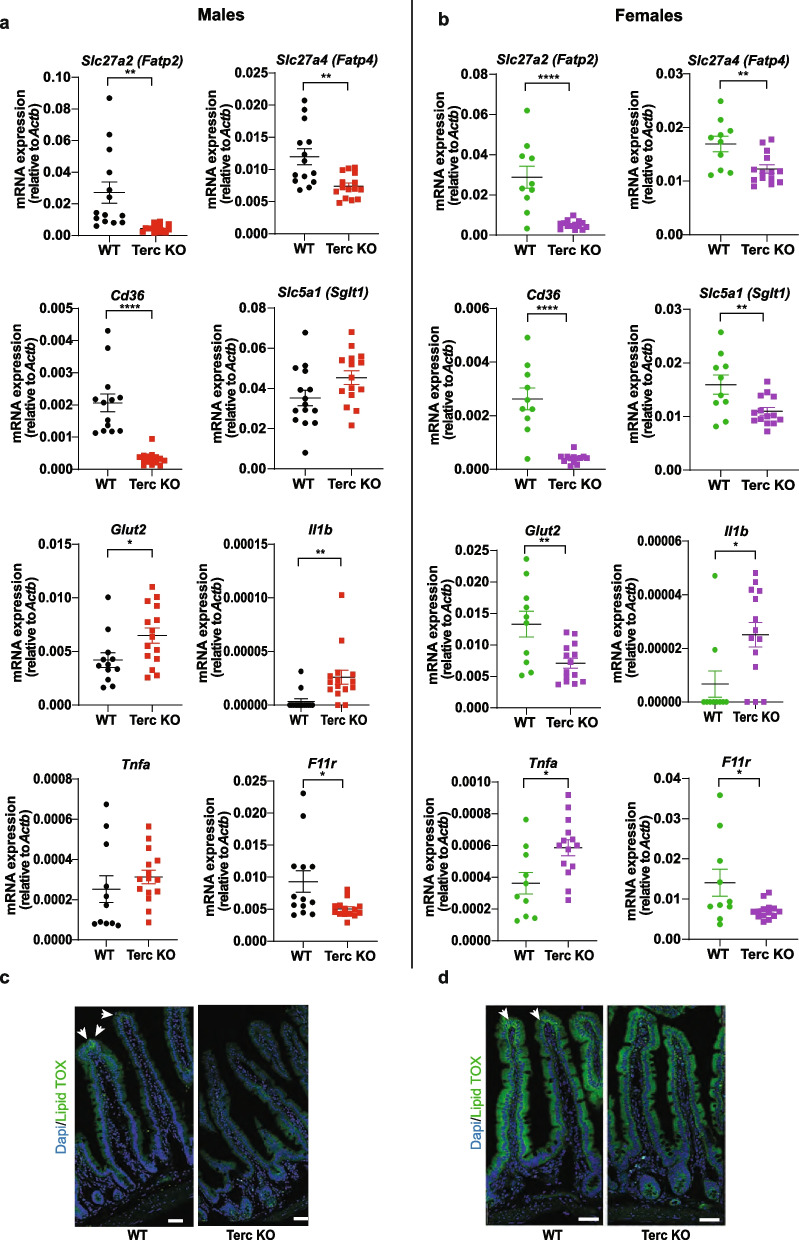
Telomere shortening reduces expression of intestinal fatty acid uptake and transport related genes. qPCR analysis of expression of fatty acid uptake, glucose uptake, inflammation and tight junction related genes in 14 month old male (**a**) and female (**b**) G2-Terc.^−/−^ and control mice (Male mice: *n* = 12–16 WT, *n* = 14–15 KO; female mice: *n* = 10 WT, *n* = 14 KO). LipidTOX staining of lipid droplets in intestinal villi from male (**c**) and female (**d**) mice. Green: lipid droplet, blue: DAPI. Statistical analysis by unpaired *t-*test. *p < 0.05, **p < 0.01, ***p < 0.001, ****p < 0.0001. Scale bar represents 75µm

**Fig. 5:**
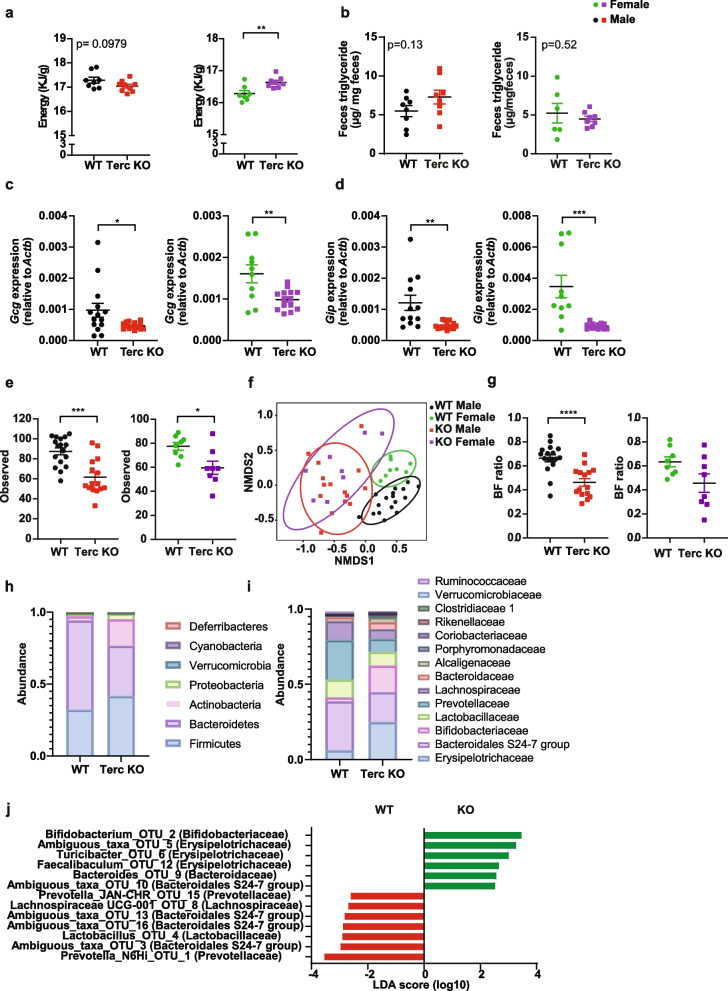
Shorter telomere length results in reconfiguration of the gut microbiome. **a** Fecal caloric energy content in 12 month old female (*n* = 7 WT, *n* = 8 KO) and male (*n* = 8 WT, *n* = 9 KO) G2-Terc^−/−^ and control mice. **b** Fecal triglyceride content of 12 month old female (*n* = 6 WT, *n* = 8 KO) and male (*n* = 8 WT, *n* = 8 KO) G2-Terc^−/−^ and control mice. Gene expression from intestinal enterocytes from male and female G2-Terc^−/−^ and control mice for (**c**) *Glucagon* (*Gcg*) and (**d**) *Gip*. Male mice: *n* = 14 WT, *n* = 15 KO; female mice: *n* = 10 WT, *n* = 14 KO. Microbiota analysis using 16S rRNA amplicon sequencing of fecal samples from 14 month old female (*n* = 8 WT and *n* = 8 KO) and male (*n* = 17 WT and *n* = 15 KO) G2-Terc^−/−^ and control mice. **e** Analysis of alpha diversity using observed species richness. **f** Analysis of beta diversity a NMDS plot. **g** Ratio of Bacteroides/Firmicutes (BF ratio). **h** Microbiota composition on phylum level. **i** Microbiota composition on family level. **j** LefSe analysis of microbiota changes. Statistical analysis of **a-b** was performed using an unpaired *t*-test and **c** and** e** by a Mann–Whitney test, *p < 0.05, **p < 0.01, ***p < 0.001, ****p < 0.0001. Red and black dots represent male mice. Green and purple dots represent female mice

Intestinal malabsorption could be a possible explanation for the reduction in body weight and energy expenditure with no changes in food intake. Villi length in the duodenum and jejunum of male G2-Terc^−/−^ mice was not different from controls (Additional file 1: Figure S2a). Fatty Acid Transport Protein 2 (Fatp2) and Fatty Acid Transport Protein 4 (Fatp4) are the main fatty acid transporters facilitating luminal free fatty acid (FFA) uptake by intestinal epithelial cells. Interestingly, male and female G2-Terc^−/−^ mice had greatly reduced (84% and 82%, respectively) intestinal epithelial expression of *Fatp2* (unpaired *t-*test male *p* = 0.0017) and 38% and 28% reduction, respectively, in Fatp4 (unpaired *t-*test male *p* = 0.0015) and *Cd36* (unpaired *t-*test male and female *p* < 0.0001, 83.92% and 84.75% reduction, respectively) (Fig. 4a and b). This was in line with strongly reduced neutral lipid stains observed in female and male enterocytes of G2-Terc^−/−^ mice compared to controls (Fig. 4c and d). Expression of the main enterocyte glucose exporter *Sglt1* was not changed in male G2-Terc^−/−^ mice, whereas *Glut2* expression was 54% upregulated in male G2-Terc−/− mice (unpaired *t-*test male *p* = 0.0042). In contrast expression of *Glut2* (unpaired *t-*test *p* = 0.0045) and *Sglt1* (unpaired *t-*test male *p* = 0.0083) were reduced by 47% and 31%, respectively, in female G2-Terc^−/−^ mice (Fig. 4a and b). In contrast to the mRNA expression, protein levels of GLUT2 were increased in enterocytes of female G2-Terc^−/−^ mice, with a similar trend in male mice (Additional file 1: Figure S2b). In addition, we tested the expression of the tight junction component *F11r*, which was downregulated in female and male G2-Terc^−/−^ mice (unpaired *t-*test male: *p* = 0.0159, 46% reduction; female: *p* = 0.0243, 50% reduction). Furthermore, expression of *Il1b* was upregulated in enterocytes of both male (unpaired *t-*test *p* = 0.0042, 66% increases) and female (unpaired *t-*test *p* = 0.0126, 28% increase) G2-Terc^−/−^ mice. Expression of *Tnfa* was only upregulated in female mice (unpaired *t-*test *p* = 0.0127, 62% increase) (Fig. 4a and b). However, F4/80 staining to visualize macrophages, did not reveal any major inflammatory infiltrates in the intestine of G2-Terc^−/−^ mice, suggesting, if at all, only a low-grade intestinal inflammation (Additional file 1: Figure S2c). These data suggest a reduced intestinal lipid absorption as well as a potentially slightly increased intestinal inflammation in G2-Terc^−/−^ mice. Indeed, total fecal caloric content was 2% increased in female G2-Terc^−/−^ mice compared to controls (unpaired *t-*test, *p* = 0.0066), whereas this was not observed in male G2-Terc^−/−^ mice (Fig. 5a) that, however, tended to have increased fecal triglyceride content (Fig. 5b). Fecal FFA content was similar between genotypes regardless of gender, with a trend for reduced FFA levels in male G2-Terc^−/−^ mice (*p* = 0.0804, Additional file 1: Figure S3a). Differences in intestinal luminal FFA content can alter incretin expression and release [22]. Therefore, we examined the expression of Glucagon (*Gcg*) and Glucose-dependent insulinotropic polypeptide (*Gip*) genes in intestinal enterocytes. We found significantly reduced expression in male and female G2-Terc^−/−^ mice (Gcg: male, *p* = 0.0287, 51% reduction; female: *p* = 0.0044, 39% reduction; Gip: male *p* = 0.0027, 61% reduction; female *p* = 0.0004, 74% reduction; Fig. 5c and d).

To this end, we hypothesized that changes in the intestinal epithelium could be linked to a reconfiguration of the gut microbiome. Therefore, we performed microbiota composition analysis of fecal samples from male mice (15 G2-Terc^−/−^ and 17 controls) and female mice (8 G2-Terc^−/−^ and 8 controls) using 16S rRNA amplicon sequencing [23]. The analysis of alpha diversity, i.e., the complexity of the microbial community within a sample, revealed that male G2-Terc^−/−^ mice had a reduced richness (Fig. 5e, Mann–Whitney test, *p* = 0.0001) of the microbiota as well as a reduced Shannon diversity (Additional file 1: Figure S3b, Mann–Whitney test, *p* = 0.0031) in comparison to the WT mice. Female G2-Terc^−/−^ mice had a similar reduction in richness (Fig. 5e, Mann–Whitney test, *p* = 0.02), but the decrease in Shannon diversity did not reach a significant level, potentially due to the smaller sample size (Additional file 1: Figure S3b). The analysis of beta diversity, i.e., the similarity of the microbial community between samples, indicated a clear genotype-driven separation between G2-Terc^−/−^ and WT mice (Permutational Multivariate Analysis of Variance Using Adonis, PERMANOVA, R2 = 0.30542, Fig. 5f). Notably, differences were already detectable on the phylum level as highlighted by altered Bacteroides/Firmicutes ratios (Mann–Whitney test, *p* = 0.0001) in male mice and a similar trend observed in female mice (Mann–Whitney test, *p* = 0.1304, Fig. 5g). Moreover, we noted a strong expansion of the Actinobacteria phylum in G2-Terc^−/−^ mice (Fig. 5h). Specifically, increased relative abundances of the families *Bifidobacteriaceae* and *Erysipelotrichaceae* were observed in G2-Terc^−/−^ mice, while the families *Prevotellaceae* and *Muribaculaceae* (previously referred to Bacteroidales group S24-7) were decreased (Fig. 5i). A more detailed analysis of the microbiota changes using LefSe identified several operational taxonomic units (OTUs) from the above-mentioned families that were significantly enriched in G2-Terc^−/−^ mice, including for instance a specific OTU from the genus Bifidobacteria (OTU2) and the genus *Faecalibacterium* (OTU12) (Fig. 5j). Of interest, these bacteria were previously associated to health benefits, specifically to improve insulin sensitivity, influence fat mass loss, and the production of short-chain fatty acids [24–26].

## Discussion

Here we show that moderate shortening of telomeres in second generation C57BL/6 Terc^−/−^ mice improves insulin sensitivity and glucose tolerance in both male and female mice. However, this effect does not appear to be a consequence of direct effects on cellular glucose utilization, as previously suggested [21]. We did not observe any differences in systemic energy expenditure. We find that male and female G2-Terc^−/−^ mice show reduced intestinal epithelial expression of fatty acid transporters, lower intestinal epithelial triglyceride content and a reconfiguration of gut microbiota, with elevated levels of Actinobacteria. Members of this family show positive effects on various metabolic parameters. For example, *Bifidobacterium longum* APC1472 improves glucose tolerance in obese mice and lowered fasting glycemia in obese/overweight individuals [27]. Furthermore, supplementation of obese individuals with probiotics containing *Bifidobacteria* resulted in decreased body weight and improvement in wellbeing [28]. Thus, the improvement in glucose tolerance and insulin sensitivity in G2-Terc^−/−^ mice likely results from the reduced fat mass coming from a combination of reduced intestinal lipid absorption and the expansion of metabolically beneficial microbiota. Importantly, these effects are seen in both male and female mice. However, we cannot exclude that moderately increased lipid utilization of organs such as the skeletal muscle also contribute to the observed phenotype. We did not observe changes in OxPhos proteins in TA or liver in either male or female mice. Hepatic and TA triglyceride levels were unaltered in male G2-Terc^−/−^ mice. In contrast, in female G2-Terc^−/−^ mice the liver triglyceride levels were reduced, whereas they trended towards an increase in the tibialis anterior.

In addition, we observed other sex-specific differences in individual metabolic and molecular parameters. Most prominently female Terc^−/−^ mice only showed an improved glucose tolerance when high doses of glucose (4 g/kg) were administered, whereas this effect was already observed at 2 g/kg in male Terc^−/−^ mice. The most obvious explanation for this difference is the overall higher glucose tolerance and insulin sensitivity of female compared to male mice [29–33]. This is also reflected by the serum triglyceride levels. Moreover, female Terc^−/−^ mice showed increased fecal caloric content, as expected from reduced lipid uptake, whereas this was not observed in male mice. Male mice on the other hand had a tendency to increased fecal triglyceride levels. Curiously, mRNA levels of *Glut2* in enterocytes differed between male and female G2-Terc^−/−^ mice, while protein levels were significantly increased in female Terc^−/−^ mice and tended to be increased in male Terc^−/−^ mice. This indicates, as suggested in [21] that enterocytes from Terc^−/−^ mice have an increased glucose uptake capability. In principle, intestinal glucose absorption is very efficient [34, 35]. However, the observed changes in gut microbiota could mobilize otherwise inaccessible carbohydrates, compensating to some extend for the reduced lipid absorption [36]. The elevated RER during the active phase (dark cycle), in female Terc^−/−^ mice, demonstrates increased carbohydrate oxidation. This suggests that female mice per se have more efficient glucose absorption than males, which is further increased upon Terc knockout. Thus, impaired lipid absorption could result in slightly different compensatory mechanisms between male and female mice.

We did not observe a direct effect of Terc deficiency in our aged G2-Terc^−/−^ model on pancreatic beta cell function, as this was described for G4-Terc^−/−^ mice [20], although intestinal epithelial *Gcg* and *Gip* expression were significantly reduced in both genders. To this end, we conclude that the effects on beta cell function and survival observed upon strong shortening of telomeres reflects a rather indirect consequence of the overall severe pathology strongly impairing, among other cell types, intestinal and hematopoietic cell function. However, beyond the effects on glucose and insulin homeostasis, we observed additional phenotypes, such as the reduced intestinal gene expression of *F11r* and increased expression of inflammatory markers. *F11r* deficiency in mice leads to increased intestinal epithelial permeability and an increase in inflammation [37]. Intestinal permeability increases with aging [38]. Thus, it is tempting to speculate that this is directly linked to telomere length. However, we did not observe differences in macrophage infiltration or other histological abnormalities in G2-Terc^−/−^ mice. Thus, the observed changes in inflammatory gene expression do not appear to indicate a strong intestinal inflammation. However, increased telomere shortening could further promote this inflammatory phenotype.

## Conclusions

In summary, increased telomere shortening, either through genetic impairment in telomerase activity, as described here, or via environmental effects appears to have beneficial effects on glucose homeostasis, which, on first sight, contradicts many epidemiological studies in humans. However, while describing a principle mechanism, our data should not be translated one to one to humans. Humans live in a much less controlled environment, easily compensating the potential beneficial effects of reduced intestinal lipid absorption through increased food intake or substitution with carbohydrate rich foods. Moreover, further decrease in telomere length later in life, or as seen in studies using a higher number of Terc^−/−^ inbred generations, will cause various other organismal damages, eventually worsening among others, glucose homeostasis. Direct measurements of glucose and lipid absorption, as well as microbiota transfer experiments should be performed in future large-scale studies in mice or specifically designed clinical studies to proof causality of our findings. These studies will also define the transition points from beneficial to detrimental effects of telomere shortening on lipid absorption and systemic glucose homeostasis.

Nevertheless, our data unravel the principle role of telomere shortening in the regulation of systemic glucose metabolism and insulin sensitivity. This will educate future murine and human aging studies and provides important insights into the age associated development of type 2 diabetes and metabolic syndrome. Thus, moderate telomere shortening in mice reduces body weight, improves metabolic homeostasis and alters gut microbiota composition.

## Methods

### Animal husbandry

All animal experiments were approved by the Animal Ethics Committee of the government of Upper Bavaria (Germany). Mice were housed in a specific pathogen free facility at 20–23 °C and 45–60% humidity with a 12-h light /12-h dark circle in ventilated cage. Cages were supplemented with nesting material and changed every week. All mice had ad libitum access to water and a standard laboratory chow diet (Altromin, Germany). Male and female Terc^±^ mice were bought from Jackson lab (Jackson stock: 004132, Jackson laboratory, Bar harbor, Maine, USA; RRID:IMSR_JAX:004132). Terc^±^ mice were initially mated with C57BL/6N mice. Terc^±^ mice from these matings were then bred with each other. Offspring from these breedings were used to establish first generation wild type (WT; Terc^+/+^) and Terc KO (Terc^−/−^) mouse lines. Offspring from these inbreedings produced second generation Terc^−/−^ (G2- Terc^−/−^) and WT mice that were used for experiments.

### Glucose tolerance test (GTT)

Mice were fasted for 4 h during the light phase. Two types of GTTs were performed. Oral GTT (oGTT) where mice were orally gavaged with 20% glucose (Braun, Germany) (2 g/kg) and an intraperitoneal GTT (i.p. GTT) where mice were intraperitoneally injected with 20% glucose solution (2 g/kg). Blood glucose levels were measured from tail tip punctures using a Freestyle Freedom Lite glucometer (Abbott, Chicago, Illinois, USA) at the 0, 15, 30, 60, 90 and 120 min time points.

### Oral glucose-stimulated insulin secretion (oGSIS)

Mice were fasted for 8 h prior to oral gavage of 4 g/kg glucose during the light phase. The blood glucose concentrations were measured in blood collected from the tail at 0 and 2, 5, 15 and 30 min time points after gavage by using a FreeStyle Freedom Lite glucometer. Blood was collected from the tail into Microvette tubes (Sarstedt, Germany) at the same time points. Plasma was prepared by centrifugation (10 min at 10,000 × g at 4 °C) and insulin concentrations were determined using an Ultra-Sensitive Mouse Insulin ELISA Kit (CrystalChem, USA).

### Insulin tolerance test (ITT)

Mice were fasted for 4 h during the light phase and intraperitoneally injected with 0.75 (female mice) or 1.5 (male mice) IU/kg insulin (Actrapid, Novo Nordisk, Denmark). Blood glucose levels were measured from tail tip punctures using a Freestyle Freedom Lite glucometer (Abbott) at the 0, 15, 30, 60, 90 and 120 min time points.

### Pyruvate tolerance test (PTT)

Mice were fasted overnight for 16 h. 20% Pyruvate solution was prepared in Dulbecco´s phosphate buffered saline (PBS) (Gibco, USA). Mice were intraperitoneally injected with 2 g/kg pyruvate and blood glucose levels were measured at 0, 15, 30, 60, 90, 120 min time points using Freestyle Freedom lite glucometer (Abbott).

### Energy metabolism studies

These studies were conducted as previously described [39]. In brief, lean and fat mass were measured using a magnetic resonance whole-body composition analyzer (EchoMRI, Houston, Texas, USA). Food intake, water intake, respiratory exchange ratio (RER), energy expenditure (EE) and locomotor activity were measured continuously in 10-min intervals, after a 24 h acclimatization period, using indirect calorimetry (TSE Phenomaster, Berlin, Germany).

### Glycosylated hemoglobin (HbA1c) measurement

HbA1c was measured from random fed mice during the light phase without fasting. HbA1c was measured using a DCA Vantage Analyzer (Siemens, Munich, Germany).

### Pancreatic islets isolation

Mice were sacrificed by cervical dislocation. To perfuse the pancreas, the bile duct was clamped and injected with 1 ml of ice cold 1 mg/ml collagenase P (Roche, Switzerland) solution in G-solution (HBSS (Lonza, Switzerland) with 1% BSA (Sigma-Aldrich, USA). The perfused pancreas was consequently dissected and placed into a 15 ml Falcon tube with 1 ml of collagenase P solution for 15 min at 37 °C. Strong shaking was performed in the middle of incubation. Then, 12 ml of cold G-solution was filled into the falcon tubes with samples, and this was followed by centrifugation at 1,620 rpm at room temperature (RT). Pellets were washed with 10 ml of G-solution. After washing with G-solution, the pellets were re-suspended in 5.5 ml of 15% Optiprep solution (5 ml of 10% RPMI 1640 (Lonza, Switzerland) which diluted by G-solution + 3 ml of 40% Optiprep (Sigma-Aldrich) per sample), and placed on top of 2.5 ml of the gradient solution. To form a three-layer gradient, 6 ml of G-solution was layered on the top. Samples were then incubated for 10 min at RT before centrifugation at 1,700 rpm. Finally, the interphase between the upper and the middle layer of the gradient was harvested, filtered through a 70 μm Nylon filter and washed with G-solution. Islets were hand-picked under the microscope and cultured in RPMI 1640 medium overnight.

#### Ex vivo* GSIS*

Isolated islets were used for ex vivo glucose stimulated insulin secretion test after an overnight recovery in RPMI 1640 medium. Ten islets were placed in a filter basket located in each well of a 24-well cell culture plate and incubated in KRB buffer with 2.8 mM glucose for 1 h. The islets were washed with glucose-free KRB buffer one time. Subsequently, the islets were incubated for 45 min in KRB buffer with 2.8 mM glucose, supernatant was collected and stored at -80 °C for later insulin content measurement. Afterwards, islets were wash with glucose-free KRB buffer, the islets were further incubated for 45 min in KRB buffer with 16.7 mM glucose, supernatants were collected and stored in -20 °C for later insulin content measurement. Then the islets were incubated with cold acid ethanol (Ethyl Alcohol (100%): HCl (12N) = 49:1) overnight at -20 °C. The next day supernatants were collected for total insulin content measurement.

### Histology and imaging

#### Paraffin-embedded sections

Liver, perigonadal fat (pgWAT), subcutaneous fat (subWAT), duodenum, jejunum and ileum were isolated from mice and fixed with 4% paraformaldehyde (PFA) (Roth, Germany) overnight at 4 °C. The tissues were dehydrated into ascending concentrations of ethanol (70–100%), cleared by xylene then embedded in paraffin (Leica, Germany). Two micrometer tissue sections were cut using a semi-automated microtome (Leica, Wetzlar, Germany) and the sections were stained with a hematoxylin and eosin (H&E) stain as previously described [40]. Imaging was done using ECLIPSE Ci microscope (Nikon, Minato city, Tokyo, Japan).

#### Cryo-embedded sections

Pancreas was isolated from mice and incubated with 4% PFA (Roth, Germany) overnight at 4 °C and washed twice with PBS. Afterwards, pancreas was cryoprotected in a sequential gradient of 7.5% and 15% sucrose for 1 h at RT then it was incubated with 30% sucrose overnight at 4 °C. Subsequently, pancreas was incubated with 30% sucrose and Tissue-Tek O.C.T compound (Sakura, Japan) in a 1:1 solution for 1 h at RT. In the end, tissues were embedded into a Tissue-Tek cryomold (Sakura, Osaka, Japan) with Tissue-Tek O.C.T compound on dry ice and stored at -80 °C. Sections of 10 μm thickness were cut using a cryostat (Leica, Wetzler, Germany), mounted on a SuperFrost Adhesion Microscopic slides (Epredia, Portsmouth, New Hampshire, USA) and dried for 10 min at RT then stored at − 20 °C. H&E staining and sections imaging were done as described above.

#### Immunofluorescence staining and imaging

Cryo-sections were washed 3 × 15 min with PBS and permeabilized in 0.5% TritonX-100 in PBS for 20 min, washed with PBS and blocked for one hour in blocking solution (0.1% Tween-20, 10% FCS, 0.1% BSA and 3% donkey serum). Sections were incubated with the primary antibody overnight at 4 °C. All antibodies were diluted with blocking solution. Sections were washed 4 × with PBS-0.1% Tween-20 and one time with PBS on a shaker and incubated with secondary antibodies and DAPI, diluted with blocking solution for 2 h at room temperature. After additional washing in PBST and PBS, sections were mounted with fluorescence mounting medium (DAKO, S3023), dried in the dark overnight and stored at 4 °C. Following antibodies and compounds were used: Rat monoclonal anti-F4/80 (1:100, Abcam Cat# ab6640, RRID:AB_1140040), HCS LipidTOX Green (1:400, Life technologies, catalog H34475), Cy3-Donkey anti Rat (1:800, Jackson ImmunoResearch Labs Cat# 712–165-153, RRID:AB_2340667). All images were acquired with a Leica SP8 confocal microscope using LAS AF software (Leica). Images were analyzed using ImageJ software (win64,1.53c).

#### Enterocytes isolation

This protocol is a modified version of an already published protocol [41]. Small intestine was dissected and flushed with ice cold PBS. For histology the first 2 cm of the small intestine, following the stomach, were considered as duodenum and the last 2 cm before the caecum as ileum. For the jejunum we collected 2 cm of tissue in the middle section of the small intestine.

The remaining small intestine pieces were cut open and washed in ice cold PBS. Intestinal pieces were incubated in reagent 1 (30 mM ethylenediaminetetraacetic acid (EDTA), 1 mM dithiothreitol (DTT) in PBS) for 30 min on ice. The intestine was then incubated in reagent 2 (30 mM EDTA in PBS) for 10 min at 37 °C followed by shaking to dissociate the enterocytes from the basement membrane. The enterocytes were pelleted by centrifugation at 1,000 × g for 5 min at 4 °C and frozen at -80 °C until use.

#### DNA extraction for telomere length quantification

One volume of phenol: choloroform: isoamyl alcohol (25:24:1) (Roth, Germany) was added to the sample followed by centrifugation for 5 min at 16,000 × g at room temperature (RT). The upper phase was collected. One microliter of 20 mg/ml glycogen (Serva, Germany), 7.5 M ammonium acetate (0.5 × sample volume) and 100% Ethanol (2.5 x (sample volume + ammonium acetate volume)) were added to the sample. The sample was then incubated at -20 °C overnight. The DNA was pelleted by centrifugation at 4 °C for 30 min at 16,000 × g. The supernatant was removed and 150 µl of 70% ethanol was added to the sample followed by centrifugation at 4 °C for 30 min at 16,000 × g then the supernatant was discarded. This step was repeated once and the sample was left to dry at RT for 10 min. The sample was reconstituted in water.

#### RNA extraction, real time PCR

Total RNA was extracted from isolated enterocytes using RNeasy Mini Kit (Qiagen, Germany) following the manufacturer’s instructions. Complementary DNA (cDNA) was reverse transcribed from isolated total RNA using High-Capacity cDNA Reverse Transcription Kit (Thermo Fisher Scientific, USA) following the manufacturer´s instructions. Real time PCR (qPCR) was performed using iTaq Universal SYBR green Supermix (Bio-Rad, USA) in CFX384 Touch Real-Time PCR Detection System (Bio-rad). Expression of genes of interest was normalized to that of β-actin (*Actb*) gene. Differential gene expression levels were calculated using the ∆Ct method. Glucose transporter 2 (*Glut2),* Interleukin-1b *(Il1b),* Tumor necrosis factor alpha *(Tnfa), Sodium-dependent glucose transporter (Slc5a1/Sglt1), Cd36,* Fatty acid transport protein 2 *(Slc27a2/Fatp2),* Fatty acid transport protein 4 *(Slc27a4/Fatp4), F11 receptor (F11r),* Glucagon* (Gcg),* Glucose-dependent Insulinotropic polypeptide* (Gip)* expression were measured.

#### Western blot

Enterocytes, liver and tibialis anterior (TA) were homogenized using a TissueLyzer II (Qiagen, Germany) twice for 2 min at 30 Hz in lysis buffer consisting of RIPA buffer (50 mM Tris pH = 7.4, 150 mM NaCl, 1 mM EDTA, 1%TritonX100), 1% protease inhibitor (Sigma-Aldrich, USA), 1% phosphatase inhibitor cocktail II (Sigma-Aldrich) and 1% phosphatase inhibitor cocktail III (Sigma-Aldrich). 0.1% sodium dodecyl sulfate (SDS) was added to the lysates after homogenization. Samples were centrifuged for 15 min, 14000 g at 4 °C. Supernatant was collected and protein concentration was measured using Pierce BCA Protein Assay Kit (Thermo Fisher Scientific, USA). Enterocyte samples were then boiled in NuPAGE™ LDS Sample Buffer (4X) (Thermo Fisher Scientific), β-mercaptoethanol (final conc. 2.5%) and water for 5 min at 95 °C. Liver and TA samples that were used for OxPhos protein detection were heated for 10 min at 37 °C. Samples were loaded on a 10% sodium dodecyl sulfate polyacrylamide gel. Samples were plotted on a 0.45 µm PVDF transfer membrane. Membranes were either incubated with Rabbit anti-GLUT2 antibody (samples from female,1:1000; samples from male, 1:3000. Sigma-Aldrich Cat# 07–1402-I) followed by goat anti-Rabbit-HRP secondary antibody (1:2000, Thermo Fisher Scientific Cat# 31,460, RRID:AB_228341), mouse anti-β-actin-HRP (1:500, Santa Cruz, Cat# sc-47778). or Mouse anti-OxPhos antibody cocktail (1:1000, Invitrogen, Cat# 45–8099, RRID:AB_2533835) followed by goat anti-Mouse-HRP secondary antibody (1:5000, Invitrogen Cat# A16066, RRID:AB_2534739), rabbit anti-Pyruvate Dehydrogenase (PDH) (1:1000, cell signaling, Cat# 3205, RRID:AB_2162926) followed by goat anti-Rabbit-HRP secondary antibody or mouse anti-GAPDH (1:5000, Merck, Cat# CB1001; RRID:AB_2107426) followed by goat anti-Mouse-HRP secondary antibody. Bands were developed by addition of Immobilon Western Chemiluminescent HRP Substrate (Merck Millipore) and detected using Bio Rad ChemiDoc MP Imaging System (RRID:SCR_019037). Band intensity was analyzed using Fiji (RRID:SCR_002285).

#### Telomere length quantification

Using isolated enterocyte DNA, telomere length relative to *Actb* was determined using the Absolute Mouse Telomere Length Quantification Qpcr Assay Kit (ScienCell, USA).

#### Serum, liver, tibialis anterior and feces metabolites

Serum was prepared by centrifugation for 10 min at 10,000 × g at 4 °C. Insulin concentration was determined by Ultra-Sensitive Mouse Insulin ELISA Kit (CrystalChem, USA). The frozen liver and feces stored at -80 °C were powered under liquid nitrogen. Triglyceride content in liver, TA, serum and feces were measured according to the steps of the Triglyceride Quantification Colorimetric/Fluorometric kit (BioVision, USA).

#### Fecal caloric content measurement

Feces was collected over a three-day period. Feces was dried at 65 °C for 3 days along with samples of diet. Energy content of feces and diet was measured using a C7000 Calorimeter (Ika, Staufen, Germany).

#### Microbiota measurement and analysis

Fecel pellets were collected from the colon during sacrifice and initially kept on dry ice. Long-term storage was at -80 °C without addition of preservatives. DNA was extracted using the ZymoBIOMICS 96 MagBead DNA Kit (Zymo Research Europe GmbH, Freiburg, Germany). In brief, samples were homogenized in lysis buffer by bead beating three times for 5 min (MiniBeadBeater 96) followed by DNA isolation according to the manufactures instruction on a Tecan Fluent liquid handling platform. DNA was normalized and the hypervariable region V4 of the 16S rRNA (F515/R806) gene was amplified in accordance with previously described protocols (Caporaso JG et al., 2011). Amplicons were sequenced on the Illumina MiSeq platform (PE300) and reads were cut at 250 bp. The mean counts per sample were 37432.646 ± 4749.619. The Usearch11 software package (https://drive5.com/usearch/) was used to assemble (-fastq_mergepairs –with fastq_maxdiffs 30), quality control, and cluster obtained reads. Quality filtering was set up with the fastq_filter (-fastq_maxee 1); minimum read length, 250 bp. Reads were clustered into 97% ID operational taxonomic units (OTUs). The UPARSE algorithm (Edgar RC 2013) was used to determine the OTU clusters and representative sequences. The Silva database v138 (RRID:SCR_006423) (Quast C et al., 2012) and the RDP Classifier (Wang Q et al., 2007) were used for taxonomic assignment with a bootstrap confidence cut-off of 80%. OTUs with an abundance < 0.02% were pruned. Resulting OTU absolute table was used further for statistical analyses and data visualization in R package phyloseq (McMurdie PJ et al., 2013).

### Statistics

All statistical analyses were done using GraphPad Prism 8 (RRID:SCR_002798). Data are shown as mean ± standard error of the mean (SEM). Statistical significance was calculated using unpaired student´s *t-*test or, in case of multiple comparisons, one- or two-way analysis of variance (ANOVA) followed by turkey´s or Sidak’s multiple comparison´s test. Values with p-value lower than 0.05 (*p* < 0.05) were considered statistically significant.

## Supplementary Information

**Additional file 1:** **Figure S1.** Telomere shortening and metabolic characterization of young G2-Terc^-/-^ mice. Body weight and body composition of 9-week-old maleand femaleG2-Terc^-/-^ and control mice.Triglyceride measurement of liver and tibialis anteriorof 14-month-old male and female G2-Terc^-/-^ and WT mice. Raw data are shown in Additional file 3: Table S1b.Western blot for OxPhos proteins of 14-month-old male and female G2-Terc^-/-^ and WT mice. Uncropped western blots are shown in Additional file 2: Figure S4a&b. Gene expression of *Il6*and *Tnfa*from pgWAT of 14-month-old male G2-Terc^-/-^ and WT mice. Percentage of glycosylated hemoglobinof 9-week-old maleand femaleG2-Terc^-/-^ and control mice. All data are shown as mean ± SE. Statistical analysis using unpaired *t-*tests. *p<0.05, **p<0.01, ***p<0.001, ****p<0.0001. **Figure S2.** Intestinal characterization of G2-Terc^-/-^ mice.Intestinal villi length of the duodenumand jejunumof 14 months old male G2-Terc^-/-^ and control mice. Raw data are shown in Additional file 3: Table S1c.GLUT2 protein expression in intestinal enterocytes from male and female G2-Terc^-/-^ and control mice. *n*=8 WT, *n*=9 KO. Red and black dots represent male mice. Green and purple dots represent female mice. Uncropped western blots are shown in Additional file 2: Figure S4c.F4/80 immunofluorescence staining.male andfemale mice. Red: F4/80, and blue: DAPI. Scale bar represents 75 µm. All data are presented as mean ±SE. a and b were analyzed using unpaired *t-*tests. *p<0.05, **p<0.01, ***p<0.001, ****p<0.0001. **Figure S3.** Reduced telomere length results in a reconfiguration of the gut microbiome.Free fatty acids content in feces from male and female G2-Terc^-/-^ and control miceShannon diversity analysis using 16S rRNA amplicon sequencing of fecal samples from 14-month-old femaleand maleG2-Terc^-/-^ and control mice. All data are presented as mean ±SE. Data were analyzed using unpaired *t-*tests. *p<0.05, **p<0.01, ***p<0.001, ****p<0.0001. Red and black dots represent male mice. Green and purple dots represent female mice.

**Additional file 2:** **Figure S4.** Uncropped western blots. Uncropped western blots ofliver Oxphos complex,Tibialis anterior and GLUT2 in G2-Terc^-/-^ mice and controls.

**Additional file 3**.

## Data Availability

All data generated or analysed during this study are included in this published article, its supplementary information files and publicly available repositories.16srRNA data are deposited in the NCBI repository (Accession no. PRJNA966188).

## Abbreviations

ANOVA: Analysis of variance
BAT: Brown adipose tissue
Cdna: Complementary DNA
DTT: Dithiothreitol
EE: Energy expenditure
EDTA: Ethylenediaminetetraacetic acid
F11r: F11 receptor
FATP2: Fatty acid transport protein 2
FATP4: Fatty acid transport protein 4
G4-Terc^−^^/^^−^: Fourth generation Terc Knockout mice
Gcg: Glucagon
Glut2: Glucose transporter 2
Gip: Glucose-dependent insulinotropic polypeptide
HbA1c: Glycosylated hemoglobin
H&E: Hematoxylin and eosin
ITT: Insulin tolerance test
Il-1b: Interleukin-1b
i.p. GTT: Intraperitoneal glucose tolerance test
OUT: Operational taxonomic unit
oGTT: Oral glucose tolerance test
oGSIS: Oral glucose-stimulated insulin secretion
Oxphos: Oxidative phosphorylation
PFA: Paraformaldehyde
pgWAT: Perigonadal white adipose tissue
PBS: Phosphate buffered saline
PTT: Pyruvate tolerance test
qPCR: Real time PCR
RER: Respiratory exchange ratio
RT: Room temperature
G2-Terc^−^^/^^−^: Second generation Terc Knockout mice
SDS: Sodium dodecyl sulfate
Slc5a1/Sglt1: Sodium-dependent glucose transporter
SEM: Standard error of the mean
ScWAT: Subcutaneous white adipose tissue
Tert: Telomerase reverse transcriptase
Terc: Telomerase RNA component
Terc KO: Terc^−^^/^^−^
TA: Tibialis anterior
Tnf-α: Tumor necrosis factor alpha
Actb: β-Actin
