# Supplementary material for: Reduced intestinal lipid absorption improves glucose metabolism in aged G2-Terc knockout mice
Source: BMC Biol. 2023 Jul 4;21:150. doi: 10.1186/s12915-023-01629-8 (PMC10320900; doi:10.1186/s12915-023-01629-8)
Supplement: Supplementary file 1 — Additional file 1: Figure S1. Telomere shortening and metabolic characterization of young G2-Terc-/- mice. Body weight and body composition of 9-week-old maleand femaleG2-Terc-/- and control mice.Triglyceride measurement of liver and tibialis anteriorof 14-month-old male and female G2-Terc-/- and WT mice. Raw data are shown in Additional file 3: Table S1b.Western blot for OxPhos proteins of 14-month-old male and female G2-Terc-/- and WT mice. Uncropped western blots are shown in Additional file 2: Figure S4a&b. Gene expression of Il6and Tnfafrom pgWAT of 14-month-old male G2-Terc-/- and WT mice. Percentage of glycosylated hemoglobinof 9-week-old maleand femaleG2-Terc-/- and control mice. All data are shown as mean ± SE. Statistical analysis using unpaired t-tests. *p<0.05, **p<0.01, ***p<0.001, ****p<0.0001. Figure S2. Intestinal characterization of G2-Terc-/- mice.Intestinal villi length of the duodenumand jejunumof 14 months old male G2-Terc-/- and control mice. Raw data are shown in Additional file 3: Table S1c.GLUT2 protein expression in intestinal enterocytes from male and female G2-Terc-/- and control mice. n=8 WT, n=9 KO. Red and black dots represent male mice. Green and purple dots represent female mice. Uncropped western blots are shown in Additional file 2: Figure S4c.F4/80 immunofluorescence staining.male andfemale mice. Red: F4/80, and blue: DAPI. Scale bar represents 75 µm. All data are presented as mean ±SE. a and b were analyzed using unpaired t-tests. *p<0.05, **p<0.01, ***p<0.001, ****p<0.0001. Figure S3. Reduced telomere length results in a reconfiguration of the gut microbiome.Free fatty acids content in feces from male and female G2-Terc-/- and control miceShannon diversity analysis using 16S rRNA amplicon sequencing of fecal samples from 14-month-old femaleand maleG2-Terc-/- and control mice. All data are presented as mean ±SE. Data were analyzed using unpaired t-tests. *p<0.05, **p<0.01, ***p<0.001, ****p<0.0001. Red and black dots represent ma [file 12915_2023_1629_MOESM1_ESM.docx]

**Additional file 1**

**
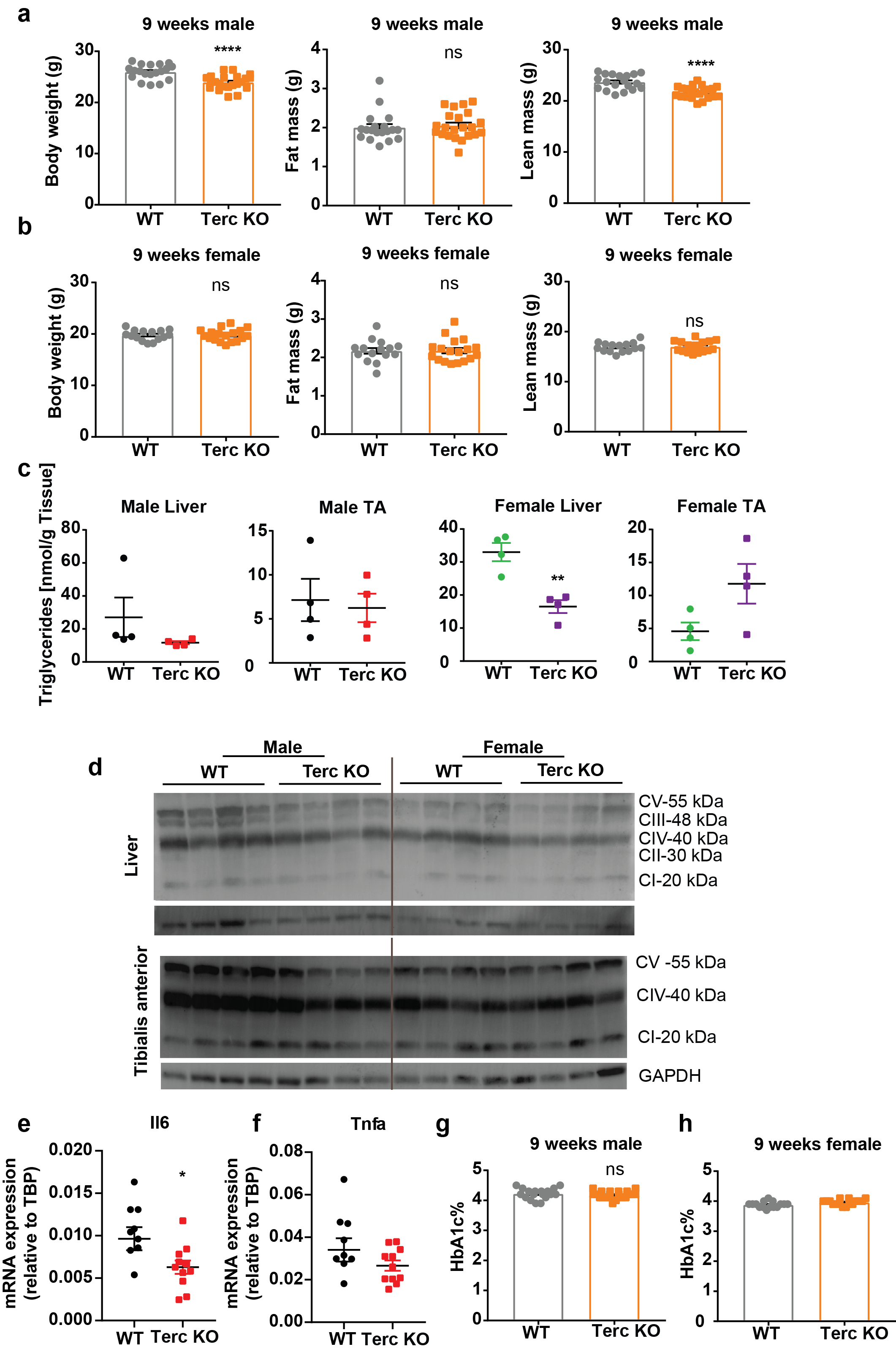
**

**Figure S1: Telomere shortening and metabolic characterization of young G2-Terc^-/-^** **mice.**

Body weight and body composition of 9-week-old male (**a**) and female (**b**) G2-Terc^-/-^ and control mice (Male, n=19 WT, n=22 KO, Female: n=15 WT, n=18 KO). (**c**) Triglyceride measurement of liver and tibialis anterior (TA) of 14-month-old male and female G2-Terc^-/-^ and wt mice (n=4). (**d**) Western blot for OxPhos proteins of 14-month-old male and female G2-Terc^-/-^ and wt mice. Gene expression of *Il6* (**e)** and *Tnfa *(**f**) from pgWAT of 14-month-old male G2-Terc^-/-^ and wt mice. Percentage of glycosylated hemoglobin (HbA1c%) of 9-week-old male (**g**) and female (**h**) G2-Terc^-/-^ and control mice (Male, n=19 WT, n=22 KO, Female: n=15 WT, n=18 KO). All data are shown as mean ± SE. Statistical analysis using unpaired *t-*tests. *p<0.05, **p<0.01, ***p<0.001, ****p<0.0001

**
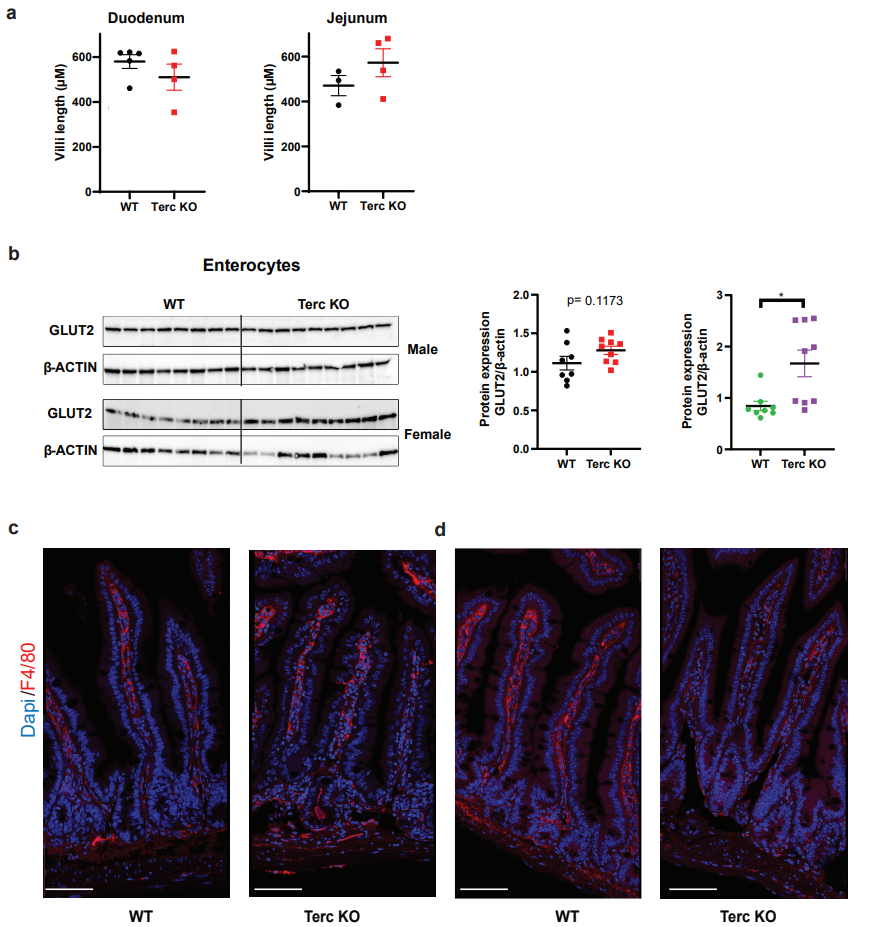
**

**Figure S2: Intestinal characterization of G2-Terc^-/-^ mice**

(**a**) Intestinal villi length of the duodenum (n=5 WAT, n=4 KO) and jejunum (n=3 WAT, n=4 KO) of 14 months old male G2-Terc^-/-^ and control mice. (**b**) GLUT2 protein expression in intestinal enterocytes from male and female G2-Terc^-/-^ and control mice. n=8 WT, n=9 KO. Red and black dots represent male mice. Green and purple dots represent female mice. (**c,d**) F4/80 immunofluorescence staining. (**c**) male and (**d**) female mice. Red: F4/80, and blue: Dapi. Scale bar represents 75 µm. All data are presented as mean ±SE. **a** and **b** were analyzed using unpaired *t-*tests. *p<0.05, **p<0.01, ***p<0.001, ****p<0.0001


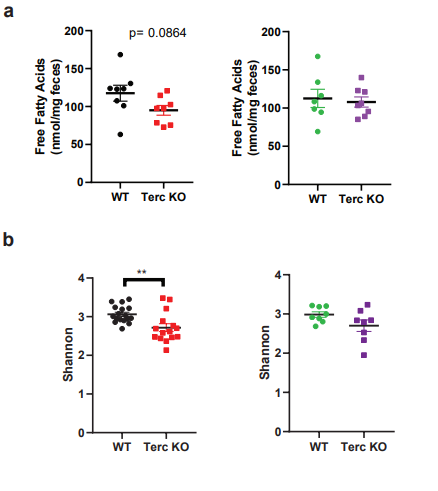


**Figure S3: Reduced telomere length results in a reconfiguration of the gut microbiome.**

(**a**) Free fatty acids content in feces from male and female G2-Terc^-/-^ and control mice (male n=8 WT and n=8 KO, female n=7 WT and n=8 KO) (**b**) Shannon diversity analysis using 16S rRNA amplicon sequencing of fecal samples from 14-month-old female (n=8 WT and n=8 KO) and male (n=17 WT and n=15 KO) G2-Terc^-/-^ and control mice. All data are presented as mean ±SE. Data were analyzed using unpaired *t-*tests. *p<0.05, **p<0.01, ***p<0.001, ****p<0.0001. Red and black dots represent male mice. Green and purple dots represent female mice.
