# Supplementary material for: Reduced intestinal lipid absorption improves glucose metabolism in aged G2-Terc knockout mice
Source: BMC Biol. 2023 Jul 4;21:150. doi: 10.1186/s12915-023-01629-8 (PMC10320900; doi:10.1186/s12915-023-01629-8)
Supplement: Supplementary file 2 — Additional file 2: Figure S4. Uncropped western blots. Uncropped western blots ofliver Oxphos complex,Tibialis anterior and GLUT2 in G2-Terc-/- mice and controls. [file 12915_2023_1629_MOESM2_ESM.pdf]

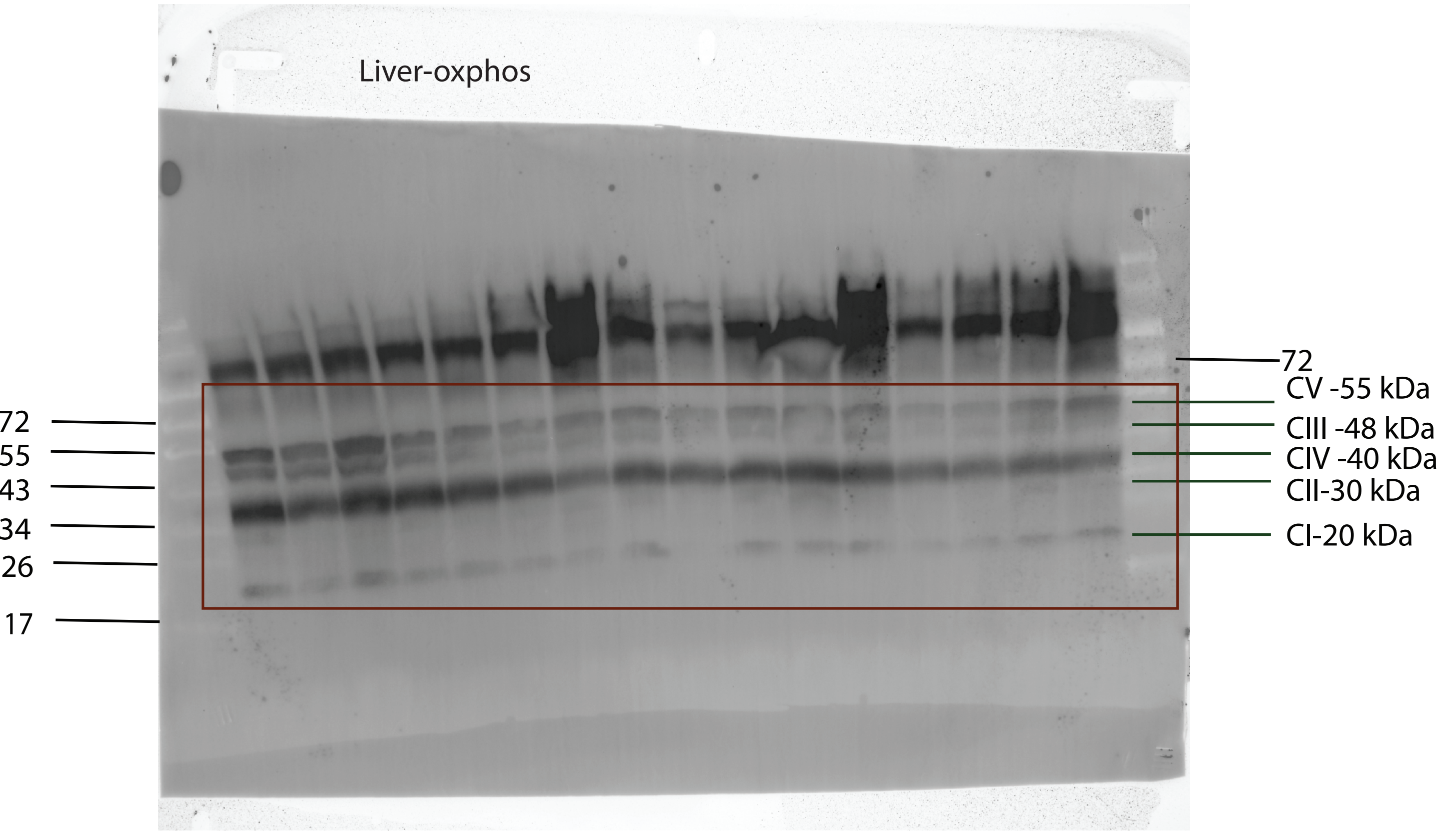
Liver-oxphos
72
55
43
34
26
17
72
CV -55 kDa
CIII -48 kDa
CIV -40 kDa
CII-30 kDa
CI-20 kDa

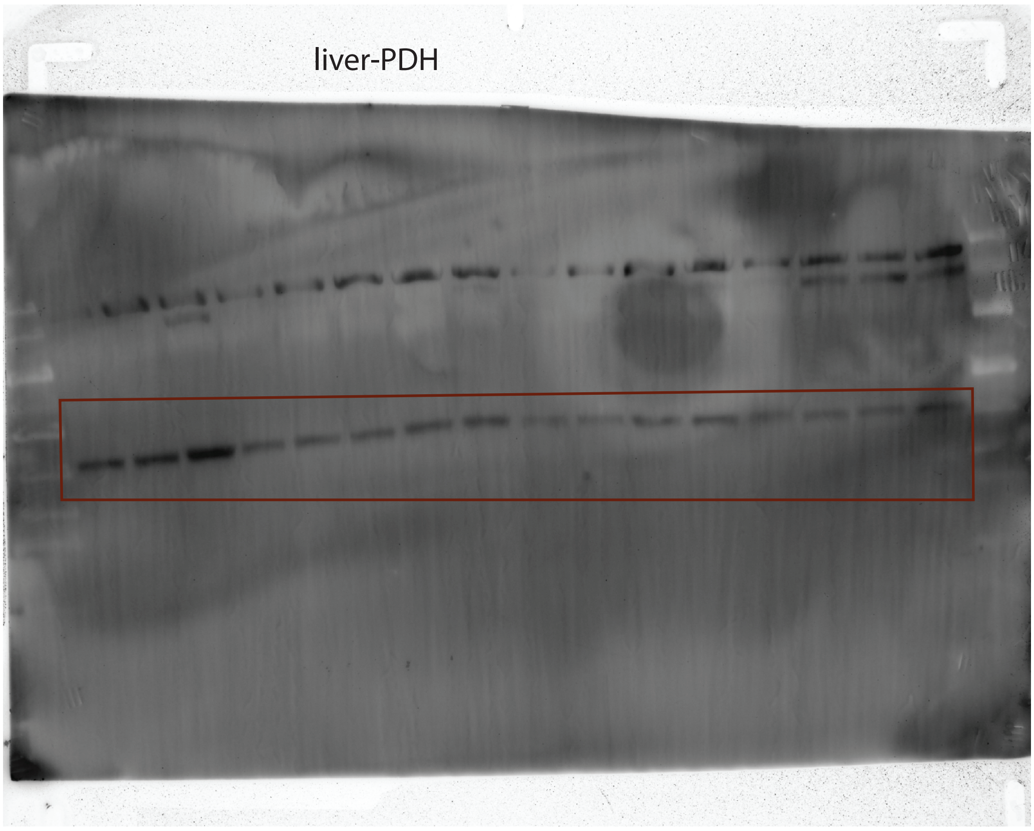
liver-PDH

Males Enterocytes

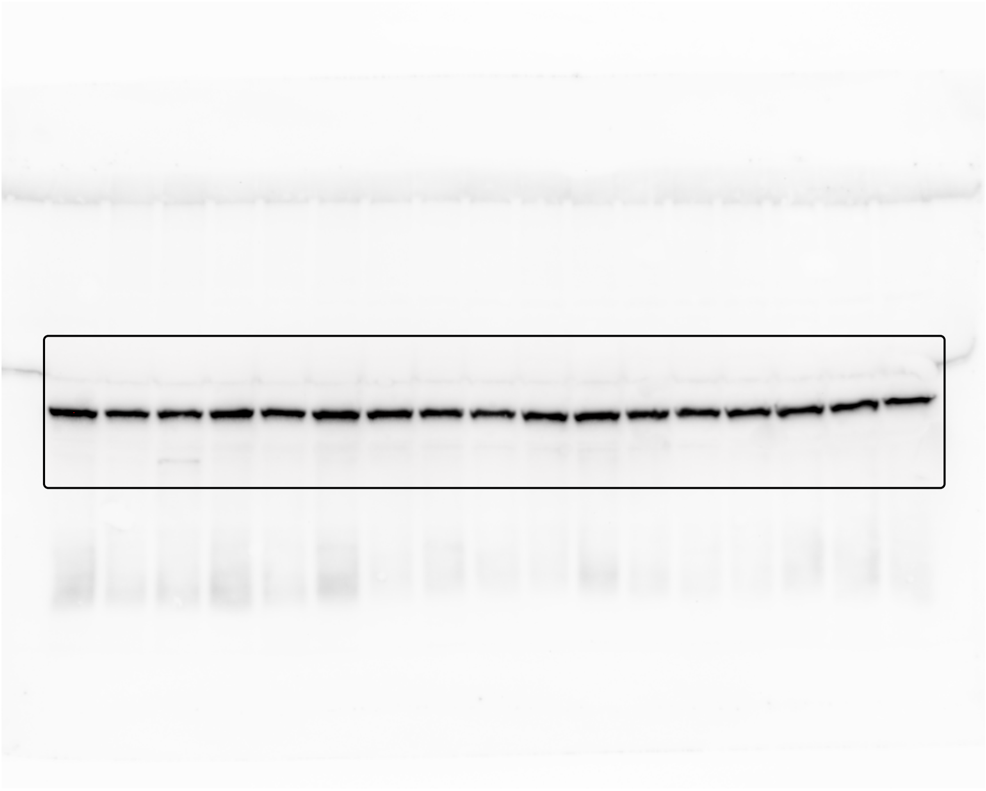

GLUT2

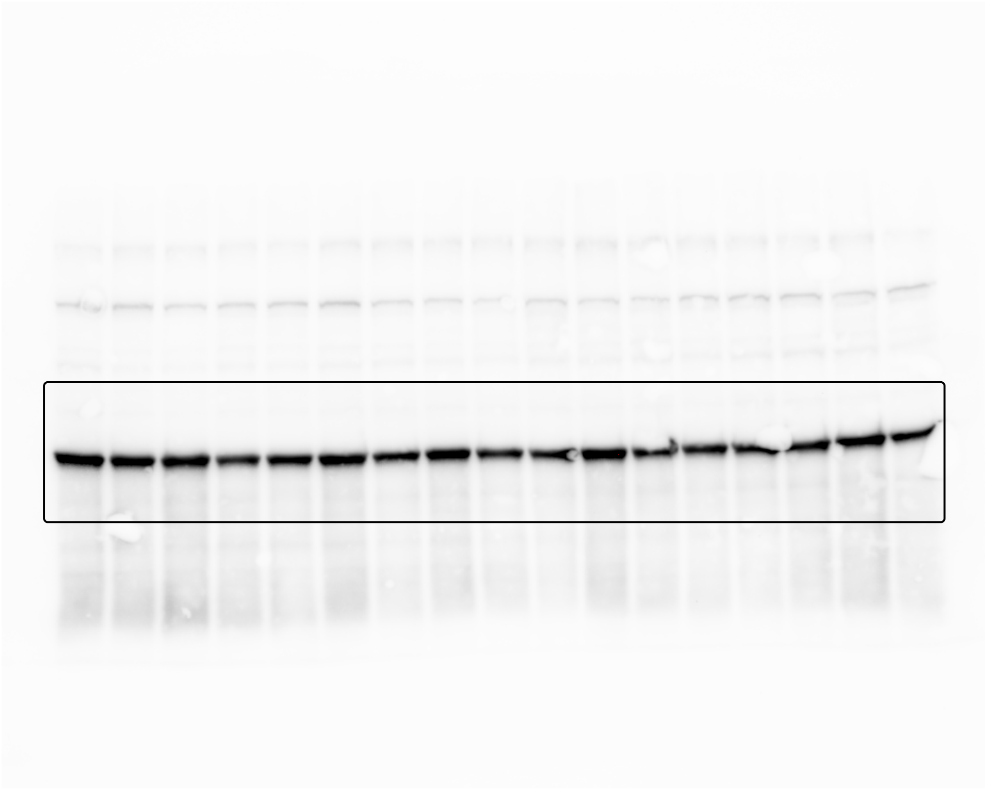

β-actin

Male and Female Tibialis Anterior

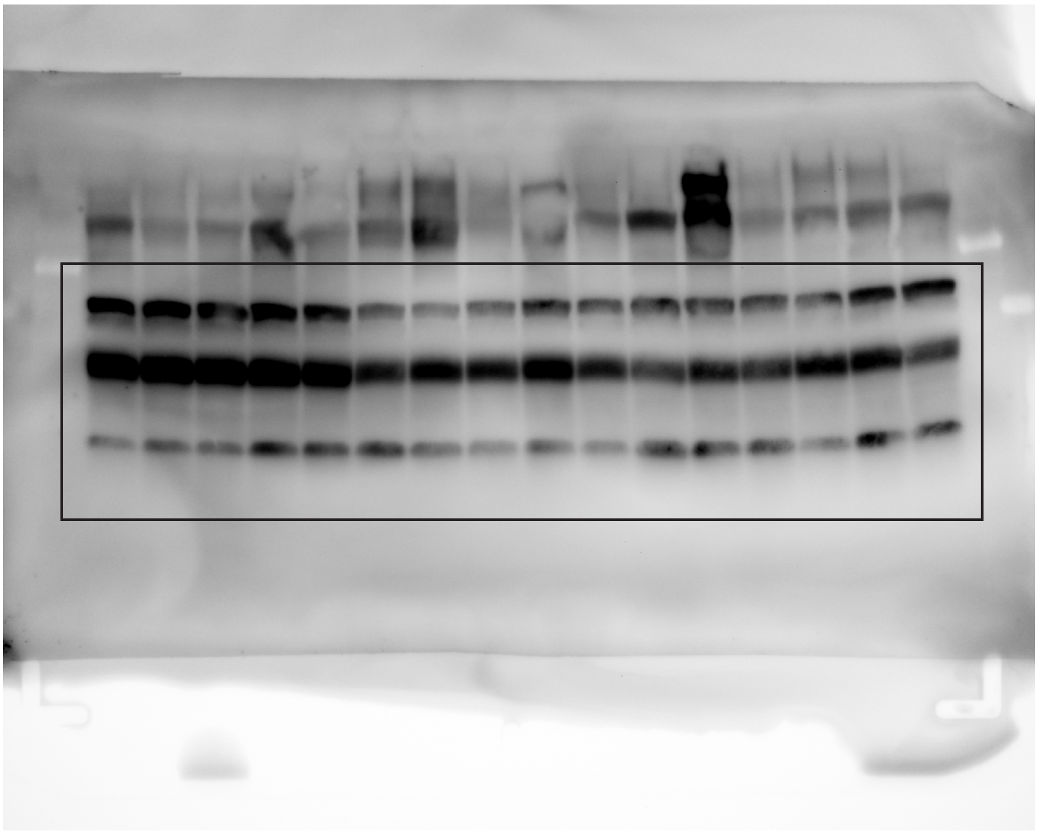

oxphos

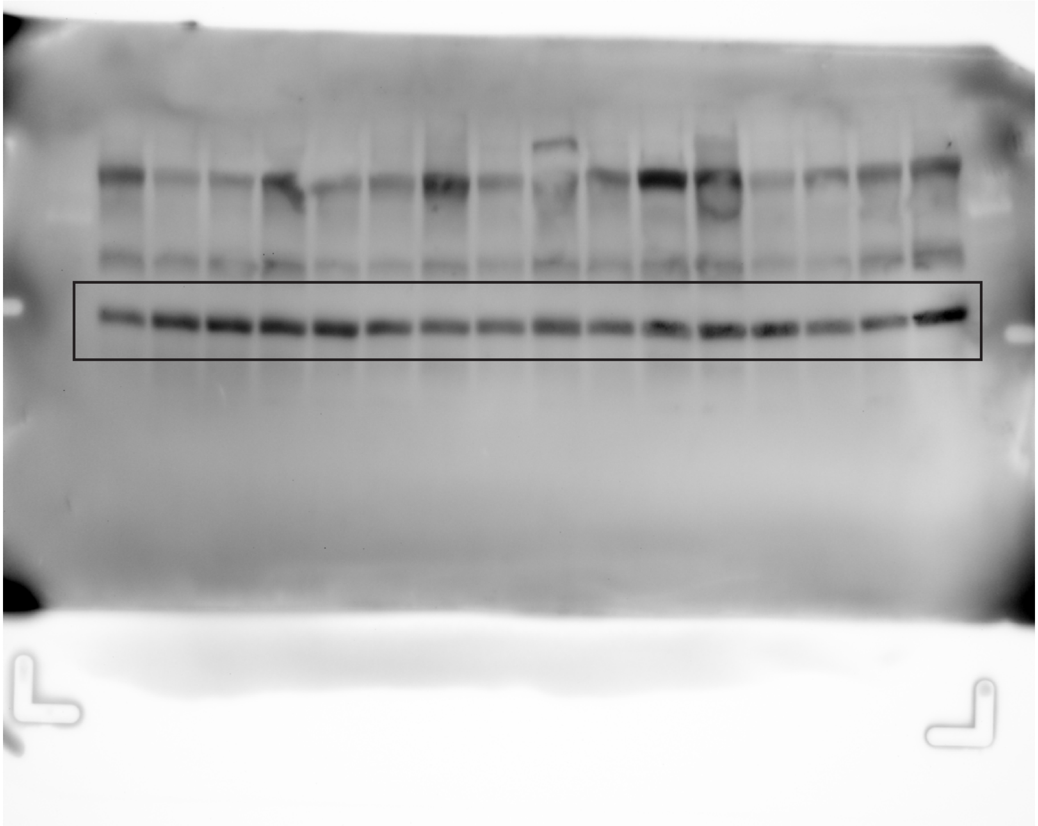

GAPDH

Female Enterocytes

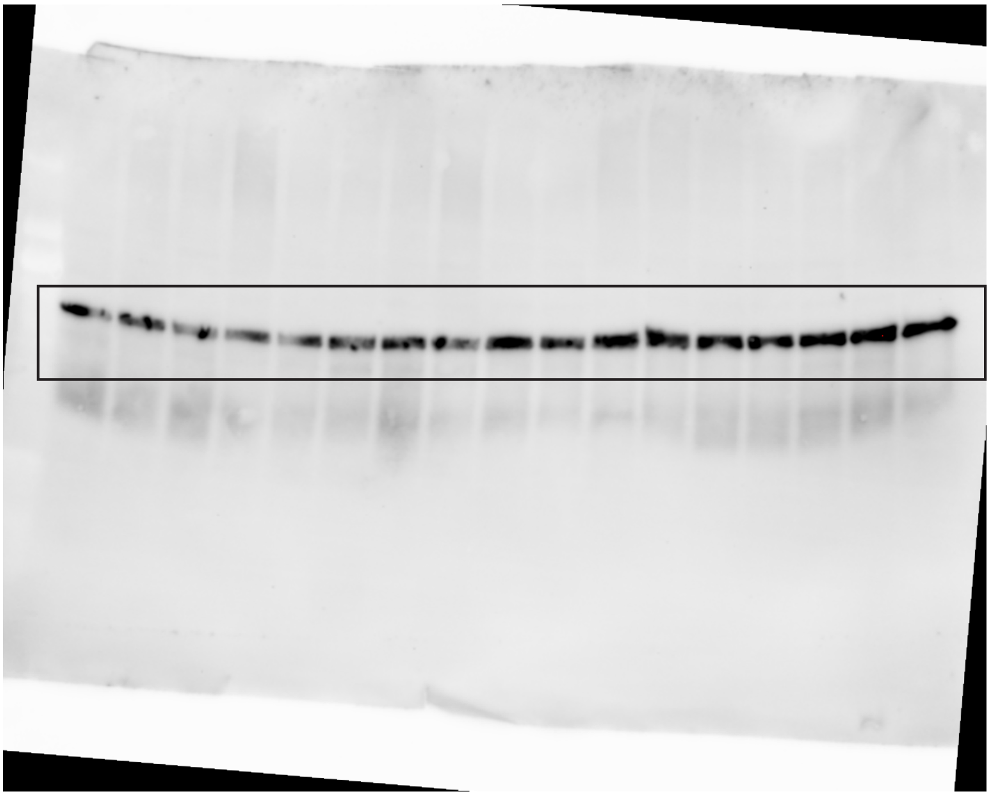

GLUT2

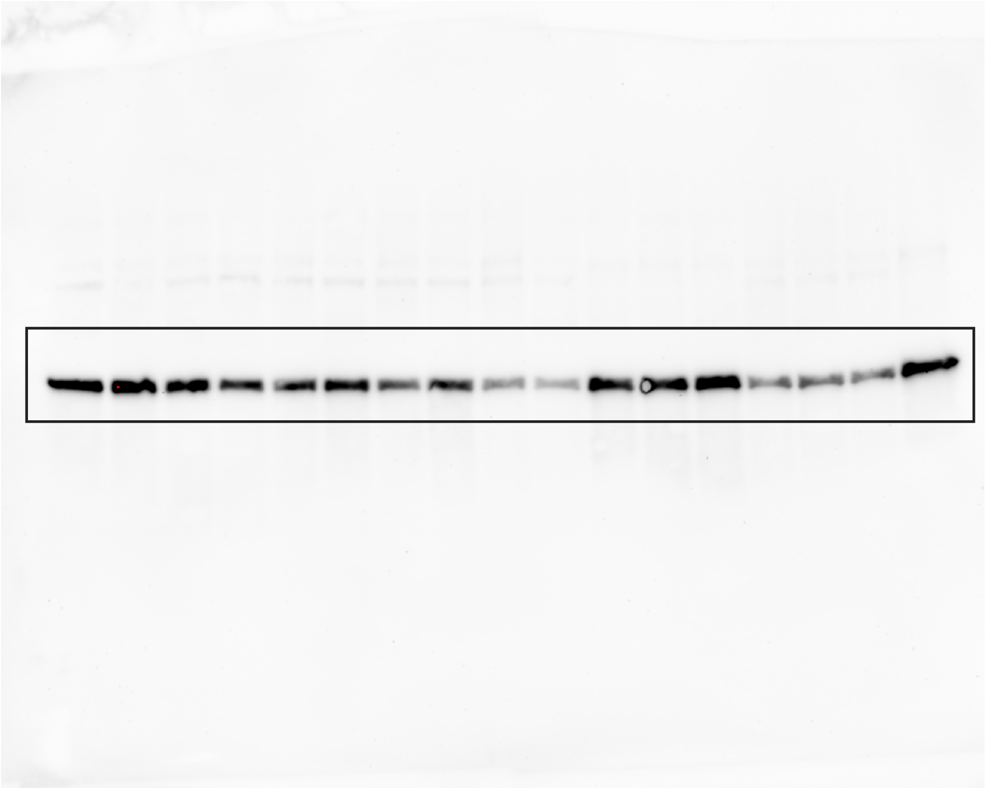

β-actin
